# A *miR-182* variant and risk of hepatocellular carcinoma in a southern Chinese population

**DOI:** 10.1186/s40246-020-00289-x

**Published:** 2020-10-15

**Authors:** Moqin Qiu, Yingchun Liu, Qiuling Lin, Zihan Zhou, Yanji Jiang, Rongrui Huo, Xiumei Liang, Xiangyuan Yu, Ji Cao, Xianguo Zhou, Hongping Yu

**Affiliations:** 1 Guangxi Medical University Cancer Hospital, Nanning, 530021 China; 2 School of Public Health, Guangxi Medical University, Nanning, 530021 China; 3 Guangxi Cancer Molecular Medical Engineering Research Center, Nanning, 530021 China; 4 Affiliated Hospital of Guilin Medical University, Guilin, 541004 China; 5 School of Public Health, Guilin Medical University, Guilin, 541004 China

**Keywords:** Hepatocellular carcinoma, *miR-182*, Polymorphism, Risk, Association

## Abstract

MicroRNAs (miRNAs) play important roles in the regulation of gene expression at the posttranscriptional level and are involved in human carcinogenesis. The aim of the current study was to investigate the associations between *miR-182* single nucleotide polymorphisms and HCC risk in a southern Chinese population. In this case-control study of 863 HCC patients and 908 cancer-free controls, we performed genotyping of *miR-182* rs4541843 and assessed its association with HCC risk. We found that individuals carrying the AG/AA genotypes of *miR-182* rs4541843 were significantly associated with an increased risk of HCC compared with those carrying the GG genotype (adjusted odds ratio (OR) = 1.71, 95% confidence interval (CI) = 1.07–2.76, *P* = 0.026). In the stratified analysis, this increased risk was more pronounced in the subgroups of older individuals (adjusted OR = 1.98, 95% CI = 1.04–3.76, *P* = 0.037), males (adjusted OR = 1.81, 95% CI = 1.09–2.99, *P* = 0.021), and never drinkers (adjusted OR = 1.84, 95% CI = 1.03–3.30, *P* = 0.041). Our results suggested that *miR-182* polymorphism rs4541843 may contribute to the susceptibility to HCC. Our findings require validation in further studies with larger sample sizes.

## Introduction

The incidence of liver cancer in China is markedly higher than that in other geographical areas around the world [1]. In 2018, approximately 841,000 people worldwide were diagnosed with liver cancer, and approximately half of these diagnoses were in China [2]. The major histological subtype of liver cancer is hepatocellular carcinoma (HCC), accounting for approximately 75–80% of all cases [3]. Several key factors may contribute to HCC, such as hepatitis B/C virus (HBV/HCV) infection, cigarette smoking, and heavy drinking, but only a small fraction of exposed individuals eventually develop HCC in their lifetimes [4]. Recently, several genome-wide association studies have identified susceptible loci harboring common single nucleotide polymorphisms (SNPs) that are relevant to the risk of HCC, suggesting that genetic factors may be responsible for susceptibility to HCC [5–8].

MicroRNAs (miRNAs) are a class of small non-coding RNAs that can regulate gene expression by pairing with the 3′ untranslated region (3′ UTR) of mRNAs [9]. To date, more than 1800 precursor miRNAs have been found in the human genome, and aberrant expression of miRNAs has been related to the etiology, diagnosis, and prognosis of many human cancers including HCC [10–12]. *MiR-182*, located in a 5-kb region of human chromosome 7q32.2, is a member of the miR-183/96/182 cluster [13]. Recently, *miR-182* has emerged as a high-priority miRNA in HCC and is involved in the development of HCC by regulating various biological processes, including cell growth, differentiation, migration, invasion, and apoptosis [14–16]. MiR-182 is frequently expressed at high levels in HCC, and its high expression is correlated with the metastasis, recurrence, and poor prognosis of HCC [17, 18].

Emerging studies have shown that SNPs in miRNAs may alter mRNA expression of their own or target genes, thus leading to tumorigenesis [19–21]. To our knowledge, there are no studies assessing the associations between *miR-182* SNPs and the risk of HCC. Given the important roles of *miR-182* in tumorigenesis, it is important to determine whether *miR-182* SNPs contribute to the susceptibility to HCC. In the current study, we conducted genotyping of *miR-182* polymorphisms and assessed their associations with HCC risk in a southern Chinese population.

## Materials and methods

### Study population

The study population consisted of 863 HCC patients who had undergone surgery between August 2007 and November 2011 in the First Affiliated Hospital of Guangxi Medical University, Affiliated Tumor Hospital of Guangxi Medical University, and First Affiliated Hospital of Guilin Medical University. The tumors were histopathologically confirmed independently as HCC by two pathologists as routine diagnosis. Patients with a prior history of other cancers, metastasized cancers, or previous radiotherapy or chemotherapy before recruitment were excluded. The 908 cancer-free controls were recruited at the same period in Southern China and were genetically unrelated and frequency matched to the cases by age (± 5 years) and sex. Participants with a history of cancer were excluded. The research protocol was approved by the Ethical Committee Review Board of Guangxi Medical University and Guilin Medical University.

With a signed written informed consent form during the interview, each subject enrolled in the study was interviewed to gather the information-related demographic data, history of tobacco and alcohol consumption, and chronic HBV infection through face-to-face interviews conducted by trained investigators. Ever smokers were defined as subjects who had smoked more than 100 cigarettes in their lifetimes; ever drinkers were defined as subjects who had used alcohol at least once a week for more than 1 year. HBV infection was defined as positive for HBV surface antigen (HBsAg). Of the 863 HCC patients, 370 HCC patients had long-term follow-up data for survival analysis. Clinical information (including AFP level, BCLC stage, cancer embolus, and cirrhosis) was collected from medical records. At 3-month intervals, follow-up information on deaths was updated by a trained clinical specialist via telephone. The survival time was calculated in months from the date of tumor resection to the date of death or last follow-up. The patients were followed up until 24 February 2020. A 5-ml peripheral blood sample was obtained from each study subject, of which 1 ml was used to determine the HBV infection status.

### SNPs selection and genotyping

The selected SNPs were screened and identified by NCBI dbSNP database (http://www.ncbi.nlm.nih.gov/) and Haploreg v4.1 (https://pubs.broadinstitute.org/mammals/haploreg/haploreg.php) in accordance with three criteria: (1) location at the *miR-182* gene region; (2) minor allele frequency (MAF) in Chinese Han population > 0.05; and (3) low linkage disequilibrium using an *r*^2^ threshold of < 0.8 for each. Ultimately, only one SNP (rs4541843 in *miR-182*) was selected in this study. Genomic DNA was extracted from peripheral blood by phenol-chloroform extraction and stored at – 80 °C. The selected SNP was genotyped using the Agena MassARRAY genotyping system (Agena, San Diego, CA) according to the manufacturer’s instructions. To ensure quality control, 10% of the samples were randomly selected to repeat the genotyping assays, and the reproducibility was 100%.

### Statistical analysis

Differences in the distributions of demographic characteristics, selected variables and frequencies of genotypes of *miR-182* rs4541843 between the cases and controls were evaluated using the *χ*^*2*^ test. Deviation of genotype frequencies in controls was tested with goodness-of-fit *χ*^*2*^ tests for Hardy-Weinberg equilibrium (HWE). We estimated the associations between *miR-182* rs4541843 genotypes and HCC risk by computing the odds ratios (ORs) and their 95% confidence intervals (CIs) with an unconditional multivariate logistic regression model. The associations between rs4541843 genotypes and HCC risk were also stratified by age, sex, smoking, drinking, and HBV infection. Hazard ratios (HRs) and 95% CIs were estimated by multivariate Cox proportional hazards model. We performed expression quantitative trait loci (eQTL) analyses with the genomic data from the genotype-tissue expression (GTEx) project to identify correlations between *miR-182* rs4541843 and mRNA expression levels of *miR-182*’s target genes. Two-sided tests of statistical significance were conducted using SPSS software, version 18.0 (SPSS Institute, Chicago, IL), and the result was considered significant when *P* < 0.05 by two-sided tests.

## Results

### Characteristics of the study population

The details of 863 HCC cases and 908 controls enrolled in this study are shown in Table 1. Because of frequency matching by design, there were no significant differences in the distributions of age or sex between the cases and controls (*P* = 0.687 and 0.053, respectively). However, cases were more likely to be smokers (43.0% vs. 25.2%, *P* < 0.001) and drinkers (38.5 vs. 21.7, *P* < 0.001) than the controls. In addition, the percentage of subjects that responded positively to HBV infection was significantly higher among the HCC cases than among the controls (84.0% vs. 9.6%, *P* < 0.001). Therefore, these variables were further adjusted in later multivariate logistic regression analyses for any residual confounding effect.

**Table 1:** Frequency distributions of selected variables in HCC cases and controls

Variables	Cases *n*(%)	Controls *n*(%)	*P* ^a^
All subjects	863(100)	908(100)	
Age			0.687
≤ 49	467(54.1)	500(55.1)	
> 49	396(45.9)	408(44.9)	
Sex			0.053
Females	100(11.6)	80(8.8)	
Males	763(88.4)	828(91.2)	
Smoking			< 0.001
Never	492(57.0)	679(74.8)	
Ever	371(43.0)	229(25.2)	
Drinking			< 0.001
Never	531(61.5)	711(78.3)	
Ever	332(38.5)	197(21.7)	
HBV infection			< 0.001
(−)	138(16.0)	821(90.4)	
(+)	725(84.0)	87(9.6)	

^a^ Two sides chi-square test

### Associations of *miR-182* rs4541843 with the risk of HCC

The observed genotype frequencies for this SNP among the control subjects were in agreement with HWE (*P* = 0.625 for *miR-182* rs4541843). After adjusted for age, gender, smoking status, drinking status, and HBV infection status, we found that the AG/AA genotypes for rs4541843 were significantly associated with an increased risk of HCC compared with the GG genotype (AG/AA vs. GG: adjusted OR = 1.71, 95% CI = 1.07–2.76, *P* = 0.026) (Table 2).

**Table 2:** Genotype frequencies of rs4541843 in cases and controls and their associations with HCC risk

Variants	Cases	Controls	*P* ^a^	Adjust OR (95% CI)	*P* ^b^
rs4541843					
GG	771	833	0.222	1	
AG	91	74		**1.71(1.06–2.74)**	**0.027**
AA	1	1		1.81(0.02–139.1)	0.789
AG/AA	92	75	0.084	**1.71(1.07–2.76)**	**0.026**

^a^Two sides chi-square test

^b^Adjusted for age, sex, smoking, drinking, and HBV infection in logistic regression models

To further identify the relationship between *miR-182* rs4541843 and HCC risk, the dominant genetic model of this SNP was stratified by subgroups of age, sex, smoking, drinking, and HBV infection. As shown in Table 3, we found that the effect of *miR-182* rs4541843 on HCC risk was more pronounced in the older group (adjusted OR = 1.98, 95% CI = 1.04–3.76, *P* = 0.037), males (adjusted OR = 1.81, 95% CI = 1.09–2.99, *P* = 0.021), and never-drinking group (adjusted OR = 1.84, 95% CI = 1.03–3.30, *P* = 0.041), while no significant interaction between *miR-182* rs4541843 genotypes and environmental factors was observed (*P* > 0.05).

**Table 3:** Stratified analysis between rs4541843 polymorphism and HCC risk

variables	rs4541843 (cases/controls)		Adjust OR (95% CI)	*P* ^a^
	GG	AG/AA	AG/AA vs. GG	
Age				
≤ 49	418/455	49/45	1.44(0.71–2.91)	0.310
> 49	353/378	43/30	**1.98(1.04–3.76)**	**0.037**
Sex				
Females	90/72	10/8	1.19(0.30–4.63)	0.807
Males	681/761	82/67	**1.81(1.09–2.99)**	**0.021**
Smoking				
Never	432/624	60/55	1.79(0.99–3.24)	0.055
Ever	339/209	32/20	1.62(0.73–3.60)	0.235
Drinking				
Never	468/655	63/56	**1.84(1.03–3.30)**	**0.041**
Ever	303/178	29/19	1.51(0.67–3.40)	0.326
HBV infection				
(−)	119/752	19/69	1.73(0.98–3.05)	0.060
(+)	652/81	73/6	1.62(0.68–3.86)	0.277

^a^Adjusted for age, sex, smoking, drinking, and HBV infection in logistic regression models

### Association between *miR-182* rs4541843 and prognosis of patients with HCC

We investigated the associations between SNP rs4541843 and the clinical features of 370 HCC patients, such as AFP level, BCLC stage, cancer embolus, and cirrhosis, but failed to find any significant associations (Table 1S). We performed a multivariate cox regression analysis on the survival of HCC patients, and the result showed that the survival of HCC patients was related to BCLC stage and cancer embolus, but not rs4541843 (Table 2S).

### Correlation between rs4541843 genotypes and mRNA expression levels of miR-182’ s target genes

Previous studies have confirmed that *miR-182* could promote HCC progression by targeting multiple genes, such as *MTSS1*, *TP53INP1*, and *RASA1* [18, 22, 23]. So, we conducted the eQTL analysis to evaluate the correlations between SNP rs4541843 genotypes and the expression levels of these genes using the data of the GTEx database. Although we found that the A allele of SNP rs4541843 appeared to be correlated with the decreased expression levels of MTSS1, TP53INP1, and RASA1 in the liver tissues, the correlations between them did not reach statistically significant level (Supplementary Figure 1).

## Discussion

In the present study, we investigated whether *miR-182* polymorphisms were associated with the risk of HCC in a southern Chinese population, and found that *miR-182* rs4541843 AG/AA genotypes were significantly associated with an evidently increased HCC risk, especially in older group, males and never-drinking group. Our findings suggest, for the first time, that genetic variant of *miR-182* might contribute to the development of HCC in this population.

*MiR-182* has been reported to act as a tumor promoter in HCC, and its upregulation has been shown to be associated with the development, metastasis, recurrence, and poor prognosis of HCC. For instance, aberrant miR-182 expression can promote HCC metastasis by targeting metastasis suppressor 1 (MTSS1), and miR-182 may be used as a diagnostic marker or potential therapeutic target in HCC [18]. Cao et al. investigated the function of *miR-182-5p* and found that the over-expression of miR-182-5p was related to poor prognosis and early recurrence in HCC. Subsequent functional experiments showed that miR-182-5p could repress FOXO3a expression by directly targeting its 3′UTR, activating the AKT/FOXO3a pathway to promote HCC progression [22]. Du et al. found that miR-182 was induced in HCC cells under hypoxia and promoted angiogenesis by targeting RASA1 [24]. Additionally, miR-182 plays an important role in drug resistance and upregulated miR-182-induced cisplatin resistance in HCC cells by regulating tumor protein 53-induced nuclearprotein 1 (TP53INP1) [23]. So, we conducted the eQTL analysis to evaluate the correlations between SNP rs4541843 genotypes and the mRNA expression levels of *miR-182*’s target genes using the data of the GTEx database. Although we found that the A allele of the SNP rs4541843 appeared to be correlated with the decreased expression levels of MTSS1, TP53INP1, and RASA1 in the liver tissues, the correlations between them did not reach statistically significant level. Tumorigenesis is a complex process, it is reasonable to speculate that *miR-182* rs4541843 might simultaneously alter the expression of multiple target genes, and subsequently contribute to the risk of HCC. However, further functional studies and association studies with larger sample sizes are warranted to confirm our findings.

Several studies have reported that single nucleotide polymorphisms in miRNA genes may influence individual susceptibility to cancers including HCC [25–27]. Xu et al. investigated the influence of *miR-146a* rs2910164 (G > C) on individual susceptibility to HCC and found that males with the GG genotype of *miR-146a* gene were 2-fold more susceptible to HCC than those carrying the CC genotype (OR = 2.02, 95% CI = 1.06–3.85, *P* = 0.034) [28]. Further investigation revealed that the GG genotype conferred a higher expression level of mature miR-146a, therefore was more susceptible to carcinogens. Liu et al. showed that the A-to-G base change in rs999885 in the promoter region of the *miR-106b-25* cluster may lead to an increased risk for HCC in HBV-persistent carriers by altering the expression of the miR-106b-25 cluster [29]. Bei et al. showed that SNP of rs1135519 could modulate miR-122 expression and contribute to the genetic susceptibility of HCC, either independently or together with rs9966765 in *miR-122* [30]. In our study, we selected one common SNP located in *miR-182* and found that the *miR-182* rs4541843 AG/AA genotypes were associated with a significantly increased risk of HCC. In the stratification analysis by variables, we observed that this significant association with the risk of HCC was particularly pronounced in the older group, males and the never-drinking group. However, studies of larger sample sizes among different populations are needed to confirm our findings.

Some potential limitations of our study should be considered. First, because our study was a hospital-based case-control study with an unavoidable selection bias, we applied a rigorous design in selecting study subjects, and controls were well matched to cases according to age and sex to minimize potential biases. Second, although the sample size of our study was large, a relatively small size in some subgroup analyses may have limited the statistical power. Finally, the precise molecular mechanism underlying the observed effects require further investigation.

In conclusion, our study provides evidence supporting the notion that the *miR-182* polymorphism might be associated with HCC risk in a southern Chinese population. Further studies with larger sample sizes and functional studies are warranted to validate our findings.

## Supplementary information

**Additional file 1:** **Table 1S.** The associations between *miR-182* rs4541843 polymorphism and clinical features of HCC patients. **Table 2S.** Cox regression analysis of the prognosis of HCC Patients.

**Additional file 2: Figure S1.** The correlation between miR-182 rs4541843 and mRNA expression of its target genes in the liver tissues from the genotype-tissue expression database.

## Data Availability

The data used to support the findings of this study are available from the corresponding author upon request.
