# Supplementary material for: A miR-182 variant and risk of hepatocellular carcinoma in a southern Chinese population
Source: Hum Genomics. 2020 Oct 15;14:38. doi: 10.1186/s40246-020-00289-x (PMC7559205; doi:10.1186/s40246-020-00289-x)
Supplement: Supplementary file 1 — Additional file 1: Table 1S. The associations between miR-182 rs4541843 polymorphism and clinical features of HCC patients. Table 2S. Cox regression analysis of the prognosis of HCC Patients. [file 40246_2020_289_MOESM1_ESM.zip › Table S1. The associations between miR-182 rs4541843 polymorphism and clinical features of HCC patients._ESM.docx]

Table 1S. The associations between *miR-182* rs4541843 polymorphism and clinical features of HCC patients

| variables | All Cases  (n=370) | rs4541843 (cases/controls) | | *P* ^a^ |
| --- | --- | --- | --- | --- |
|  |  | GG | AG/AA |  |
| Cirrhosis |  |  |  | 0.155 |
| No | 169 | 146 | 23 |  |
| Yes | 201 | 183 | 18 |  |
| AFP level (ng/mL) |  |  |  | 0.805 |
| < 400 | 219 | 194 | 25 |  |
| ≥ 400 | 151 | 135 | 16 |  |
| BCLC stage |  |  |  | 0.828 |
| A | 103 | 91 | 12 |  |
| B/C | 267 | 238 | 29 |  |
| Cancer embolus |  |  |  | 0.958 |
| No | 263 | 234 | 29 |  |
| Yes | 107 | 95 | 12 |  |

^a^ Two sides Chi-square test
