# Supplementary material for: A miR-182 variant and risk of hepatocellular carcinoma in a southern Chinese population
Source: Hum Genomics. 2020 Oct 15;14:38. doi: 10.1186/s40246-020-00289-x (PMC7559205; doi:10.1186/s40246-020-00289-x)
Supplement: Supplementary file 1 — Additional file 1: Table 1S. The associations between miR-182 rs4541843 polymorphism and clinical features of HCC patients. Table 2S. Cox regression analysis of the prognosis of HCC Patients. [file 40246_2020_289_MOESM1_ESM.zip › Table S2. Cox regression analysis of the prognosis of HCC Patients_ESM.docx]

Table 2S. Cox regression analysis of the prognosis of HCC Patients

| Variables | β | SE | HR | 95% CI | *P-*value |
| --- | --- | --- | --- | --- | --- |
| BCLC stage (A vs. B/C) | 0.575 | 0.152 | 1.78 | 1.32-2.40 | **< 0.001** |
| Cancer embolus (No vs. Yes) | 0.675 | 0.139 | 1.96 | 1.50-2.58 | **< 0.001** |
| AFP level (< 400 vs. ≥ 400) | 0.105 | 0.064 | 1.11 | 0.98-1.26 | 0.101 |
| Cirrhosis (No vs. Yes) | -0.054 | 0.126 | 0.95 | 0.74-1.21 | 0.666 |
| rs4541843 (AA/GA vs. GG) | -0.025 | 0.196 | 0.98 | 0.67-1.43 | 0.897 |
