# Supplementary figures and images for: A miR-182 variant and risk of hepatocellular carcinoma in a southern Chinese population
Source: Hum Genomics. 2020 Oct 15;14:38. doi: 10.1186/s40246-020-00289-x (PMC7559205; doi:10.1186/s40246-020-00289-x)

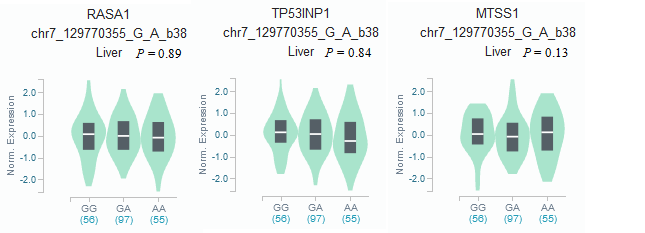

Supplement: Supplementary file 2 — Additional file 2: Figure S1. The correlation between miR-182 rs4541843 and mRNA expression of its target genes in the liver tissues from the genotype-tissue expression database. [file 40246_2020_289_MOESM2_ESM.tif]
